# MRE11-RAD50-NBS1 promotes Fanconi Anemia R-loop suppression at transcription–replication conflicts

**DOI:** 10.1038/s41467-019-12271-w

**Published:** 2019-09-19

**Authors:** Emily Yun-Chia Chang, Shuhe Tsai, Maria J. Aristizabal, James P. Wells, Yan Coulombe, Franciele F. Busatto, Yujia A. Chan, Arun Kumar, Yi Dan Zhu, Alan Ying-Hsu Wang, Louis-Alexandre Fournier, Philip Hieter, Michael S. Kobor, Jean-Yves Masson, Peter C. Stirling

**Affiliations:** 1 Terry Fox Laboratory, BC Cancer, Vancouver, V5Z 1L3 Canada; 2 Centre for Molecular Medicine and Therapeutics, BC Children’s Hospital Research Institute, Vancouver, V5Z 4H4 Canada; 3 Centre Hospitalier Universitaire de Québec-Universite Laval, Oncology Axis, Quebec City, G1R 2J6 Canada; 4 Department of Molecular Biology, Medical Biochemistry and Pathology, Laval University Cancer Research Center, Quebec City, G1V 0A6 Canada; 5 The Broad Institute of MIT and Harvard University, Cambridge, MA 02142 USA; 6 Michael Smith Laboratories, University of British Columbia, Vancouver, V6T 1Z4 Canada; 7 Department of Medical Genetics, University of British Columbia, Vancouver, V5Z 4H4 Canada

**Keywords:** Genomic instability, DNA damage and repair, Stalled forks

## Abstract

Ectopic R-loop accumulation causes DNA replication stress and genome instability. To avoid these outcomes, cells possess a range of anti-R-loop mechanisms, including RNaseH that degrades the RNA moiety in R-loops. To comprehensively identify anti-R-loop mechanisms, we performed a genome-wide trigenic interaction screen in yeast lacking *RNH1* and *RNH201*. We identified >100 genes critical for fitness in the absence of RNaseH, which were enriched for DNA replication fork maintenance factors including the MRE11-RAD50-NBS1 (MRN) complex. While MRN has been shown to promote R-loops at DNA double-strand breaks, we show that it suppresses R-loops and associated DNA damage at transcription–replication conflicts. This occurs through a non-nucleolytic function of MRE11 that is important for R-loop suppression by the Fanconi Anemia pathway. This work establishes a novel role for MRE11-RAD50-NBS1 in directing tolerance mechanisms at transcription–replication conflicts.

## Abstract (web summary)

Accumulations of R-loops can lead to genome instability. Here the authors reveal a role for the MRN complex in suppressing R-loops and associated DNA damage at transcription–replication conflicts.

## Introduction

Genome instability refers to a state in which cells experience a higher than normal mutational burden during division. Importantly, genome instability is an early driver of tumourigenesis because it increases the chance that oncogenic mutations will occur^1^. Genome instability can arise from increased DNA damage, reduced DNA replication, impaired mitotic fidelity, or perturbed cell cycle checkpoints. The interplay of these cellular pathways, their interactions with environmental insults, and their role in cancer formation, evolution, and treatment are an active area of inquiry.

One genome instability mechanism of growing importance is genotoxic transcription–replication collisions^2^. Such interference occurs normally, and cells have evolved genome architectures and replication fork-stabilizing proteins that help mitigate the risks of transcription to the genome. Nonetheless, when transcription is aberrant, or the replication fork is susceptible to stalling, collisions can lead to DNA breaks, which can promote error prone repair mechanisms and mutations. A special case of this phenomenon involves extended annealing of nascent RNA to the template DNA strand to create a three-stranded nucleic acid structure called an R-loop. R-loops perform important functions in transcriptional regulation, but can also act as barriers to replication fork progression when they accumulate ectopically through mechanisms that are not completely understood^3^.

To prevent R-loop accumulation and the resulting DNA replication stress, cells have evolved a host of R-loop degrading and mitigating strategies. For instance, splicing, RNA processing, and RNA-packaging machinery sequester nascent RNAs to prevent them from reannealing to the genome^4–6^; topological regulators such as Topoisomerase I and II restrict access of genomic DNA to RNA transcripts^7,8^; RNA nucleases including RNaseH1 and RNaseH2 specifically target the RNA moiety of R-loops^9^; Senataxin, Aquarius, and other helicases have been implicated in DNA:RNA hybrid unwinding; and DNA repair proteins such as XPF and XPG have been suggested to directly cleave R-loops to promote their resolution^10,11^. More recently, core components of the replication fork and associated factors have been linked to R-loop resolution, although the mechanisms at play are currently unclear. Notably, these include several proteins related to the Fanconi Anemia (FA) pathway, some of which have helicase activity that may remove R-loops^12–14^.

The MRE11-RAD50-NBS1 (MRN) complex is a highly conserved genome maintenance protein complex. MRN has catalytic roles in genome stability through the nuclease activity of MRE11^15^, as well as structural roles where the complex can serve as both a DNA tether and as a platform for DNA damage signaling^16^. MRN binds to DNA ends and regulates early steps of the double-strand break repair pathway, and has an important role in DNA end resection in collaboration with other nucleases^16,17^. In addition, MRN has at least two functions at stalled DNA replication forks. In BRCA2-deficient genetic backgrounds, MRE11 cleaves nascent DNA and promotes fork degradation-based restart mechanisms^18,19^. The related yeast MRX (Mre11-Rad50-Xrs2) complex functions in a nuclease-independent manner to tether sister chromatids together at stalled forks and promote efficient fork restart through an interaction with the single-stranded DNA-binding protein RPA (Replication Protein A)^20,21^.

In an effort to identify new players in cellular R-loop tolerance, we conducted a screen for genes that become essential for robust growth in yeast lacking RNaseH enzymes, which are encoded by *RNH1* and *RNH201*. We find a strong enrichment of DNA replication fork-related processes, including members of the MRX/MRN complex. In human cell models, we find that MRN functions to suppress R-loop-associated DNA damage and DNA replication stress in a manner that is independent of its nucleolytic activity. Instead, MRN is recruited to R-loop prone sites where it helps to recruit FA pathway proteins. Together, these data define a new role for MRN in coordinating the response to an endogenous source of genome instability during DNA replication.

## Results

### RNaseH-deficient yeast mutants require a robust replisome

**Fig. 1:**
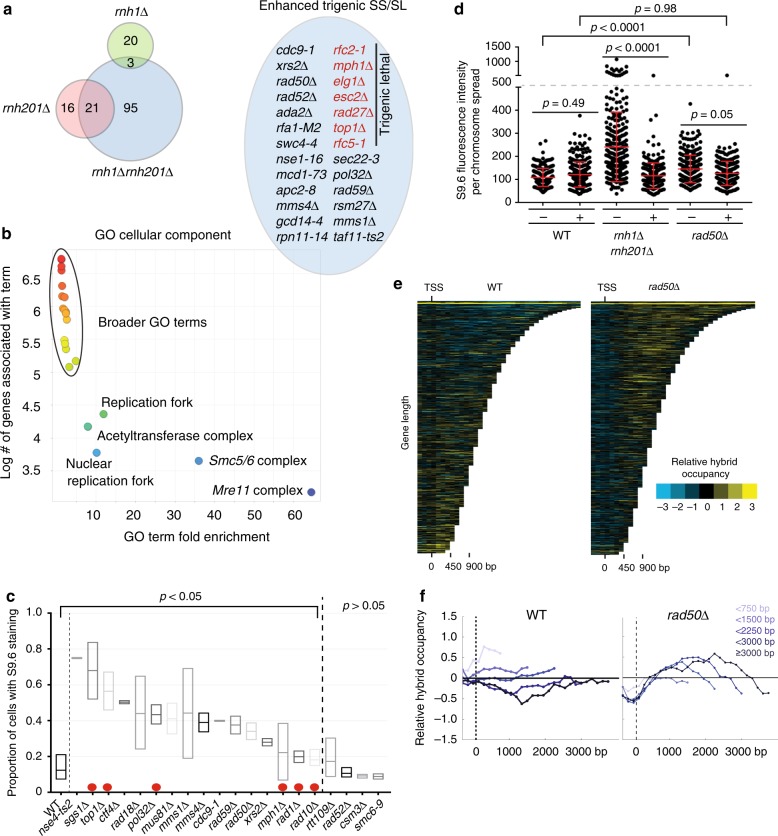
RNaseH-deficient yeast depend on replisome function and fork protection for fitness. **a** Negative genetic interaction results of double and triple mutant SGA screens with the indicated query genotype. A Venn diagram of candidate negative genetic interactions is shown (left) and validated hits that are sicker as triple mutants are in the blue circle. **b** Gene Ontology analysis of negative *rnh*ΔΔ-interacting partners. Plots show the output of ReviGO, which effectively trims redundant GO terms. **c** S9.6 staining of chromosome spreads in the indicated strains. Strains between the dotted lines had a significantly higher proportion of stained nuclei than WT (Fisher exact test. *p* < 0.05, Holm–Bonferroni corrected). Red dots indicate predicted positive controls (see main text). Box plots show the minimum and maximum values with the line at the mean value. **d** Validation of DNA:RNA hybrid increases in *rad50*Δ cells. The minus and plus signs indicate that cells were grown without or with an RNH1 overexpression plasmid. **e** and **f** Mean genome-wide relative DNA:RNA hybrid occupancy in WT, and *rad50*Δ as a function of gene length. Profiles were generated in duplicate. Quantile normalized and mean data are shown here. **e** Chromatra plots showing a heat map of DNA:RNA hybrid levels in WT, and *rad50*Δ. **f** A total of 4868 genes were split into the indicated gene length categories (538 genes < 750 bp, 1861 genes < 1500 bp, 1263 genes < 2250 bp, 636 genes < 3000 bp, and 570 genes ≤ 3000 bp) with mean enrichment scores calculated and plotted for each category

RNaseH1, encoded by *RNH1*, and RNaseH2, a trimeric complex whose catalytic subunit is encoded by *RNH201*, exert semi-redundant functions in genome maintenance^22^. Although both enzymes can digest the RNA moiety in R-loops, Rnh201 also functions in ribonucleotide-excision repair (RER) and Okazaki fragment processing, wheresa Rnh1 has mitochondrial functions^9,23^. We reasoned that mutants that specifically require the overlapping R-loop resolving activity will be less fit as triple mutants (*genex*Δ*rnh1*Δ*rnh201*Δ) in comparison with either double mutant combination (*genex*Δ*rnh1*Δ or *genex*Δ*rnh201*Δ). To conduct this experiment, we used synthetic genetic array (SGA^24^) with an *rnh1*Δ*rnh201*Δ double mutant query strain (*rnh*ΔΔ), and generated genetic interaction profiles for each double mutant and triple mutant combination (Supplementary Data 1). Our screen revealed fewer significant interactions for *rnh1*Δ (23) than for *rnh201*Δ (37), and a much larger group for *rnh*ΔΔ (119), illustrating the strong buffering relationship between Rnh1 and Rnh201 (Fig. 1a). Although a majority of *rnh201*Δ interactions were shared with *rnh*ΔΔ, there were 95 candidate negative genetic interactions unique to the *rnh*ΔΔ query (Fig. 1). We validated the genetic interactions for a selection of hits and found that although some mutants were already synthetic sick (SS) when *RNH201* was absent, many were considerably sicker or inviable when *RNH1* was also absent (Fig. 1a).

GO analysis of the raw *rnh*ΔΔ-negative genetic interaction list revealed a set of highly enriched terms entirely directed at DNA repair and DNA replication (Fig. 1b and Supplementary Fig. 1A). Highly enriched cellular components include the Mre11 complex, Smc5/6 complex and Replication Fork, whereas enriched biological processes include DNA strand elongation and intra-S DNA damage checkpoint. These suggest that losing both R-loop-mitigating enzymes in the *rnh*ΔΔ strain places a significant burden on normal replication, which is evident from increased fitness defects when replication is further perturbed. These findings cohere with recent reports characterizing the *rnh*ΔΔ strain in the presence of conditional Topoisomerase I inactivation^25^, and with the known role of R-loops in causing replication stress^3,7^.

To determine whether candidates from our genetic screen actually regulated R-loop levels, we directly tested the accumulation of DNA:RNA hybrids in 20 candidate R-loop regulatory mutant strains by staining chromosome spreads with the S9.6 antibody that targets DNA:RNA hybrids (Fig. 1c)^26,27^. This small screen included six known or predicted positive controls (red dots in Fig. 1c) selected based on studies in yeast (*SGS1*, *TOP1*, *MPH1*^8,14,28^), or of human orthologues (*RAD1/10*, *POL32*)^10,29^. All six strains displayed an increase in S9.6 staining, providing additional confidence in the assay. Collectively, this experiment determined that multiple DNA replication-associated repair factors (*NSE4*, *SGS1, TOP1, RAD18, POL32, MUS81/MMS4, RAD50, XRS2*, and *MPH1*) were both negative genetic interactors of *rnh*ΔΔ and also accumulated DNA:RNA hybrids when mutated (Fig. 1c). Such observations have only been reported for the Mre11-Rad50-Xrs2 complex in promoting break-associated R-loop formation in *Schizosaccharomyces Pombe*^30^ or in regulating aberrant replication intermediates in Senataxin-deficient yeast^31^. Therefore, we first confirmed by quantitative growth curve analysis that triple mutants of *rnh*ΔΔ and either *rad50*Δ or *xrs2*Δ had a significant loss of fitness (Supplementary Fig. 1B). *MRE11* and *RNH1* are linked genes on yeast chrXIII so triple mutants with *mre11*Δ were not made. We also confirmed that deletion of *RAD50* led to increased DNA:RNA hybrid formation by quantifying S9.6 staining in *rad50*Δ yeast with or without *RNH1* overexpression (Fig. 1d). This analysis confirmed that loss of *RAD50* increased S9.6 staining in an RNaseH1-sensitive manner.

Having detected increases in total DNA:RNA hybrid levels, we turned to genome-wide DNA:RNA immunoprecipitation followed by tiling microarray (DRIP-chip) to determine where along the genome the DNA:RNA hybrid landscape was altered upon loss of RAD50^32^. The relative DNA:RNA hybrid measures obtained from our ChIP analysis showed that loss of *RAD50* shifts DNA:RNA hybrid occupancy compared with WT cells, enriching for relative hybrid levels at long protein coding genes (Fig. 1e, f). This is similar to what we previously observed for *sgs1*Δ yeast. In fact, comparison of these data sets shows a higher correlation between the entire *rad50*Δ and *sgs1*Δ profiles compared with WT (Pearson correlation of 0.96 vs 0.92, and see additional comparisons in Supplementary Fig. 1C and Supplementary 1D). Overall our screen identifies *RAD50* as a novel regulator of DNA:RNA hybrid levels and occupancy in the yeast genome.

### MRN depletion causes R-loop-dependent DNA damage

**Fig. 2:**
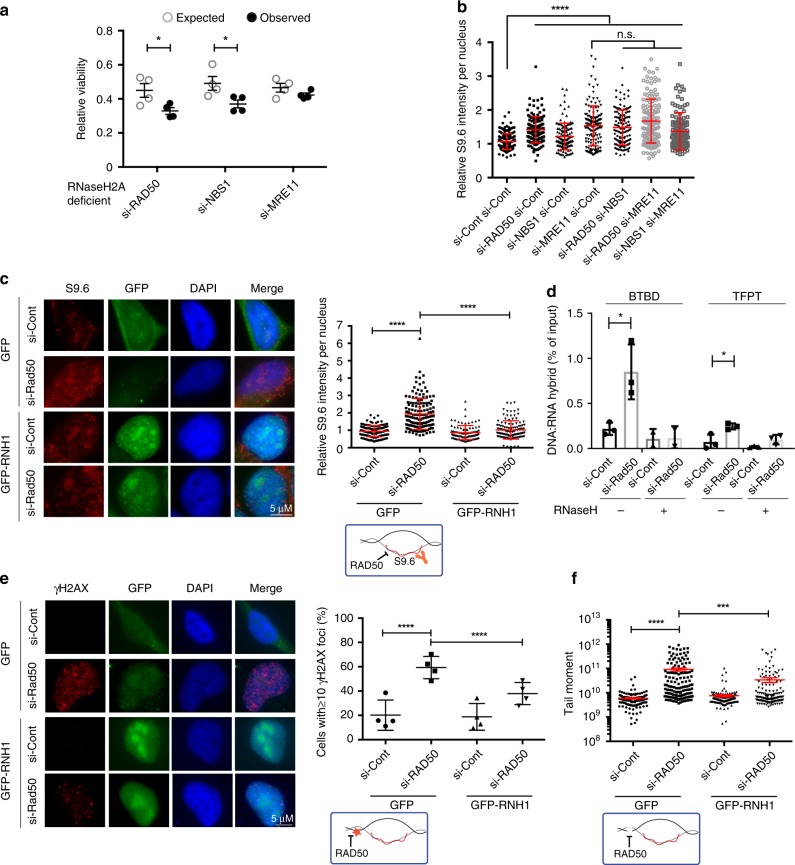
Conserved RNaseH-dependence and R-loop accumulation in human cells depleted for MRN. **a** Cell viability of RNaseH2A normal and knockout cells depleted for *RAD50*, *MRE11,* or *NBS1* as measured by crystal violet stain (*N* = 4; *P < 0.05 by *t* test; mean ± SEM) showed observed viability values were significantly lower than expected values after depletion of *RAD50* and *NBS1*. **b** Relative S9.6 staining intensity per nucleus in MRN complex depleted cells. *N* = 3; ****P < 0.0001 by ANOVA; mean ± SD. **c** Representative images (left) and quantification (right) of S9.6 staining per nucleus in HeLa cells treated with si-Cont or si-RAD50. Cells were transfected with either a control vector (GFP) or one expressing GFP-RNaseH1 (GFP-RNH1). *N* = 3; ****P < 0.0001 by *t* test; mean ± SD. **d** DRIP-qPCR analysis of R-loop accumulation at BTBD and TFPT loci in control and si-RAD50 cells with or without RNaseH treatment before precipitation. *N* = 3; *P < 0.05 by *t* test; mean ± SEM. **e** and **f** RNaseH1-dependent DNA damage phenotypes in RAD50-depleted cells shown by γ-H2AX staining and neutral comet assay for DNA breaks. **e** γ-H2AX staining (*N* = 4; ****P < 0.0001 by Fisher’s exact test; mean ± SD) and **f** neutral comet assay (*N* = 3; ***P < 0.001 and ****P < 0.0001 by *t* test; mean ± SEM). For **c**–**f** and some panels in subsequent Figs. (3b, 4a, 5a, and 6), cartoon schematics of an R-loop illustrate the effects being tested by the experiment are shown

Transcription–replication conflicts associated with R-loops have been implicated in replication stress in cancer^33–35^. The MRN complex has also been studied in multiple cancer models, where mutations or changes in gene expression of the MRN complex components have been associated with an increased propensity for malignant transformation^36–38^. Therefore, we wanted to validate that our observations in yeast were conserved to human cells in culture. We first tested whether human RNaseH2A knockout cells exhibited enhanced sickness when depleted for MRN components. The siRNA knockdown of MRE11, RAD50, or NBS1 in isogenic RNaseH2A+ or RNaseH2A− HeLa cells^39^ showed selective toxicity in the knockout cells, consistent with a conserved genetic interaction between MRN and RNaseH (Fig. 2a and Supplementary Fig. 2A). We next tested whether MRN depletion increased R-loop accumulation. S9.6 staining in cells with MRN subunit depletion showed significant increases in R-loop accumulation (Fig. 2b and Supplementary Fig. 2B), and this was suppressed by co-transfection with GFP-RNaseH1 (Fig. 2c)^40^. We further performed in vitro digestion of DNA:RNA with recombinant RNaseH and dsRNA with recombinant *RNa*seIII and confirmed that RNaseH abolished the induced S9.6 signal in Rad50-depleted cells, whereas RNaseIII reduced the dsRNA background staining without affecting the difference in S9.6 signal between control and Rad50-depleted cells (Supplementary Fig. 2C and Supplementary Fig. 2D). Indeed, DNA:RNA immunoprecipitation (DRIP) experiments also revealed an induction of hybrid occupancy at R-loop-prone loci^41^ when *RAD50* was depleted. This DRIP signal was lost when chromatin inputs were treated with RNaseH in vitro prior to immunoprecipitation, demonstrating that DNA:RNA hybrids were responsible for the signal (Fig. 2d). Co-transfection of RAD50-depleted cells with GFP-RNaseH1 reduced signal at BTBD19 but was ineffective at the TFPT locus (Supplementary Fig. 2E). Differences in RNaseH accessibility to specific loci have been noted in other models^25^. Regardless, these data show that DNA:RNA hybrids accumulate in RAD50-depleted human cells, as they do in *rad50*Δ yeast (Fig. 1).

To determine whether the R-loop accumulation we observed contributed to DNA damage, we knocked down RAD50, which ablates complex function, and tested the R-loop-dependence of DNA damage phenotypes. Significant DNA damage induction associated with RAD50 depletion was observed by quantifying DNA damage signaling by γ-H2AX staining, or measuring DNA double-strand breaks with a neutral comet assay. Importantly, both DNA damage phenotypes were partially suppressed by the overexpression of RNaseH1 (Fig. 2e, f and Supplementary Fig. 2F). Together, these data show that human cells lacking MRN function depend on RNaseH2A for fitness, and that MRN-depleted cells accumulate R-loops and R-loop-dependent DNA damage. MRN has both structural and nucleolytic roles in double-strand break repair and replication fork stability maintenance^16^. Work in *S. pombe* has shown that DSB resection, initiated by MRN, leads to transient R-loop formation at breaks that play a role in regulating homologous recombination^30^. These functions of MRN would seem to promote R-loops. However, our data suggests that MRN also represses R-loop formation. This raises the question of which of these two pathways impacted by MRN is important for R-loop tolerance and bypass?

### MRN functions at replication-associated R-loops

**Fig. 3:**
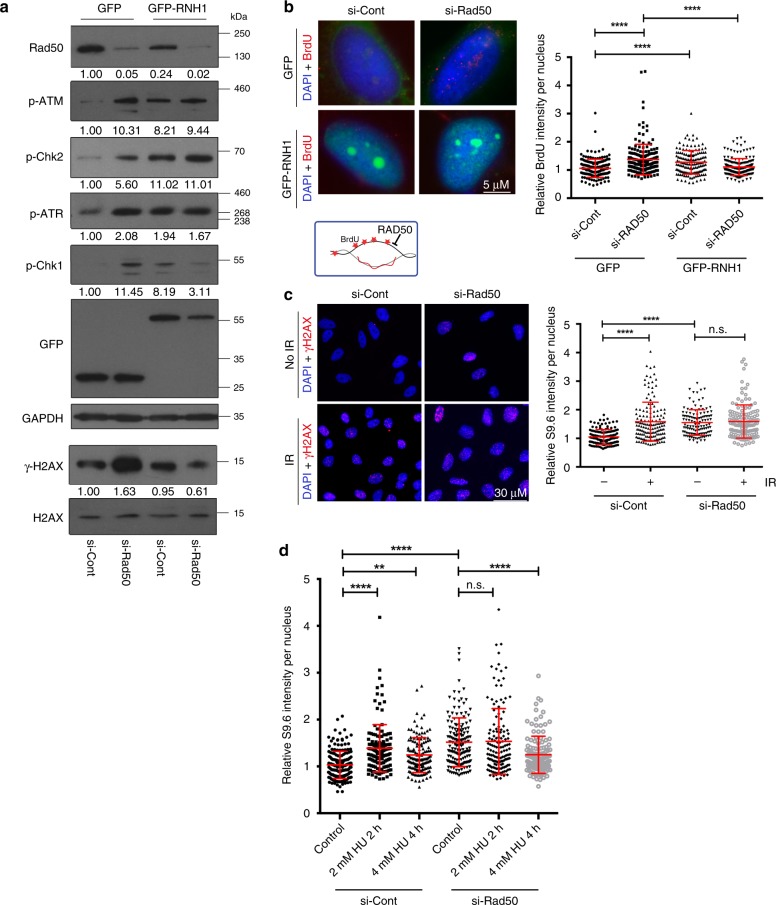
Opposing effects of RAD50 on replication-dependent and break-induced R-loops. **a** DNA damage-signaling activation. Representative western blots against the indicated epitope are shown and quantified below each panel. **b** Native BrdU staining for R-loop-dependent ssDNA exposure. Representative images (left) and nuclear fluorescence quantification are shown (right). *N* = 4; ****P < 0.0001 by ANOVA; mean ± SD. **c** Ionizing radiation induced damage and R-loops. the left panel shows γH2AX staining to confirm the damage caused by irradiation. The graph (right) shows quantification of S9.6 staining intensity. *N* = 3; ****P < 0.0001 by ANOVA; mean ± SD. **d** DNA:RNA hybrid accumulation during HU induced fork stalling or breakage. Low dose HU (2 mm) induces stalling, whereas high dose HU (4 mM) induces breakage^47^. *N* = 3; **P < 0.01 and ****P < 0.0001 by ANOVA; mean ± SD

MRN has roles at DSBs and at replication forks, and there is evidence of R-loop formation at both of these structures^7,30,42,43^. To differentiate the potential role of MRN in R-loop accumulation at these two sites, we first assessed the influence of ectopic RNaseH1 expression on the induction of DNA damage signaling in RAD50-depleted cells. RAD50 depletion activates both the ATM and ATR pathways as judged by Chk2 and Chk1 phosphorylation, respectively (Fig. 3a). As previously reported by other groups, we observed that ectopic RNaseH1 expression activated a DNA damage response (Fig. 3a)^44,45^. We hypothesize that this results from RNaseH1 mistakenly targeting the normal functions of R-loops. However, RNaseH1 expression reduced Chk1 and H2AX phosphorylation during RAD50 knockdown, indicating that the ATR-sensitive DNA replication stress response may be R-loop-dependent, but did not reduce ATM or Chk2 phosphorylation (Fig. 3a). This is consistent with recent literature, highlighting the importance of ATR signaling in mediating tolerance to replisome-R-loop conflicts^42,46^. To further explore R-loop-dependent replication stress in RAD50-depleted cells, we conducted native BrdU immunofluorescence to directly assess the exposure of single-stranded DNA, a proxy for replicative stress. RAD50-depleted cells exposed significantly more ssDNA, which was partly suppressed by RNaseH1 (Fig. 3b). Thus, R-loop-dependent replication stress may increase in RAD50-depleted cells. To determine whether RAD50 regulates R-loops at DSBs we measured irradiation induced R-loops with or without RAD50 depletion. As reported previously, irradiation of cells induced increased S9.6 staining (Fig. 3c). However, in cells depleted for RAD50, which already had higher S9.6 staining, no additional increase was observed upon irradiation (Fig. 3c). This is consistent with R-loop formation at DSBs, requiring the MRN complex to form^30^. Indeed, we further measured S9.6 signal intensity after treating cells with a low dose of HU (2 mm for 2 h), which induces fork stalling, and a high dose of HU (4 mm for 4 h), which induces DNA DSBs^47^. We confirmed these effects in our system by measuring RPA2-s33 phosphorylation as a marker of fork stalling and γ-H2AX as a marker of DSBs (Supplementary Fig. 3A and Supplementary 3B). As expected, both concentrations of HU increased S9.6 signal in control cells, suggesting that fork stalling and DNA breaks both induce R-loops formation. In cells with Rad50 depletion a high dose of HU actually decreased S9.6 signal intensity (Fig. 3d), consistent with the idea that MRN is required to generate break-induced R-loops and that fork breakage helps to resolve R-loops in this scenario^10^. Interestingly, treating RAD50-depleted cells with a low dose of HU did not change the amount of S9.6 staining, suggesting that replisome stalling may already be prevalent in siRAD50 cells (see below). These data support the model, that MRN is required for break-induced R-loop formation, and further suggest that the MRN depletion induced R-loops in unchallenged cells arise through a different mechanism.

### R-loops are barriers to replication in MRN-depleted cells

**Fig. 4:**
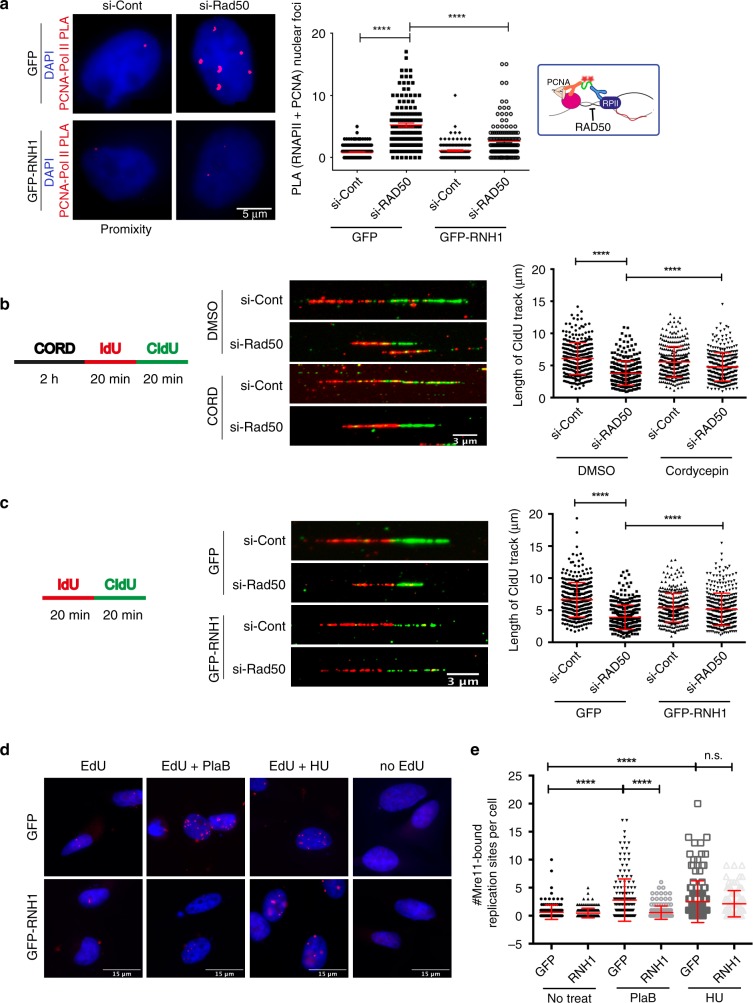
Transcription–replication conflicts impair fork progression in MRN-depleted cells. **a** Regulation of transcription–replication conflicts by RAD50. Proximity ligation assay targeting the replisome (anti-PCNA) and RNA polymerase II (anti-RNA Pol II) is shown with representative images (left) and quantification (right). *N* = 3; ****P < 0.0001 by *t* test; mean ± SEM. **b** Transcription-dependent replisome slowing in RAD50-depleted cells. Cells were treated with 50 μm cordycepin (CORD) for 2 h before IdU labeling. **c** R-loop-dependent replisome slowing in RAD50-depleted cells. For **b** and **c**, an experimental scheme (left), representative DNA fibers from the indicated conditions (middle), and quantified CldU track lengths are shown (right). *N* = 3; ****P < 0.0001 by *t* test; mean ± SD. **d** and **e** SIRF analysis of Mre11 binding to EdU-labeled nascent DNA during replication stress. PlaB or HU treatment promotes recruitment of Mre11 to forks. This recruitment is R-loop-dependent in PlaB-treated cells. Representative images (**d**), and quantification (**e**) are shown. *N* = 3 for control and PlaB, *N* = 2 for HU; ****P < 0.0001 by ANOVA; mean ± SD

In yeast, aberrant transcription–replication conflicts in *SEN1* mutant cells led to the MRX-dependent protection of forks^31^. Thus, conflicts between replication and transcription that are unresolved may lead to damage in MRN-deficient cells. To test this, we first measured the frequency of transcription–replication conflicts in MRN-depleted cells, using a proximity ligation assay (PLA) with antibodies targeting PCNA and RNA polymerase II^42^. RAD50 depletion significantly increased transcription–replication conflicts marked by PCNA-POLII PLA foci, which could be reduced by ectopic RNaseH1 expression (Fig. 4a). Next, we conducted DNA-combing experiments to directly measure replisome dynamics. As previously reported for Nbs1 and Xrs2^48,49^, *RAD50* depletion also led to a net slowing of replication progress, consistent with less processive or impaired replication, and supporting the known role of MRN in fork stabilization^21,49^. Importantly, replisome impairment was partially rescued by transcription inhibition with cordycepin, indicating that transcription impairs replication in RAD50-depleted cells (Fig. 4b). To link the replisome specifically to R-loops, we overexpressed RNaseH1, which also partially restored replication speed in RAD50-depleted cells (Fig. 4c).

If MRN has a direct role in mitigating transcription–replication conflicts associated with R-loops, it should be physically present at R-loop stalled replication forks. To detect the localization of Mre11 at replication forks during R-loop induction, we performed in situ analysis of protein interactions at DNA replication forks (SIRF)^47^ using pulsed EdU-Mre11 PLA reactions (Mre11-SIRF). Stalled forks are known to recruit Mre11^47^, so we used a low dose of HU (2 mm) as a positive control and compared the SIRF signal to cells treated with a splicing inhibitor Pladienolide B (PlaB), which is known to induce R-loops and DNA damage (Fig. 4d, e). Both PlaB and HU treatment strongly induced Mre11-SIRF signal, consistent with active recruitment of Mre11 to stalled forks^47^. Importantly, RNaseH1 overexpression reduced Mre11-SIRF signal in PlaB-treated but not HU-treated cells. Thus, impairing RNA splicing with PlaB led to R-loop stalled forks, which recruit Mre11. HU stalls replication directly by depleting nucleotides, and therefore led to Mre11-SIRF under all conditions. These data show that R-loop induction can lead to the physical recruitment of Mre11 to DNA replication fork, where MRN may play a role in promoting efficient resolution of transcription–replication conflicts.

### A non-nucleolytic role for MRN in R-loop regulation

**Fig. 5:**
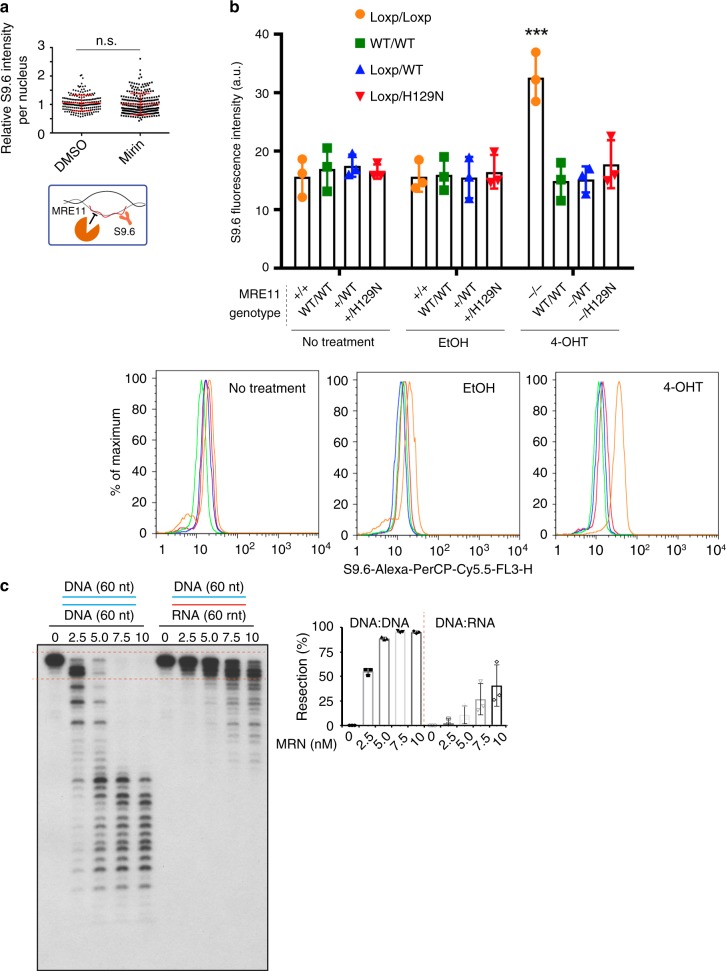
A structural role for the MRN complex in mitigating R-loop associated DNA damage. **a** Nuclear S9.6 staining intensity after treatment with MRE11 inhibitor Mirin (50 μm) is shown. *N* = 3; *t* test; mean ± SD. A schematic showing the goal of testing MRE11 nuclease activity on R-loops is show. **b** Mean S9.6 fluorescence intensity of TK6-derived lymphoblasts of the indicated genotype after treatment with EtOH (control) or 4-OHT, to induce gene knockout. Representative FACS histograms are shown below, curve colors correspond to bar graph. *N* = 3; ***P < 0.001 by ANOVA; mean ± SEM. **c** Head-to-head comparison of dsDNA and DNA:RNA hybrid resection by purified MRN complex on 60-mer duplexes. The concentration dependent degradation of the input molecules is plotted on the right

MRN has both catalytic and structural roles in promoting genome stability during DNA replication^19,21,50^. Moreover, through its interactions and roles at replication forks, MRN collaborates with many proteins potentially implicated in R-loop tolerance. For instance, studies describe MRN as recruiting helicases such as BLM^51^, binding WRN^52^, or activating the FA pathway^53^; all of which have been implicated in R-loop tolerance^12–14,54^. To determine whether the catalytic function of MRE11 is important for R-loop tolerance, we treated cells with an MRE11 inhibitor, Mirin^55^, and found no effect on S9.6 staining in cells (Fig. 5a). Furthermore, Mirin treatment did not affect the recruitment of Mre11 to R-loop prone sites as measured by ChIP-qPCR (Supplementary Fig. 3C), although Mirin did block radiation induced ATM phosphorylation as reported by Dupre et al.^55^, showing that the chemical was inhibiting Mre11 as expected (Supplementary Fig. 3D). This suggested that a structural role, rather than a catalytic role for MRN could be important for R-loop suppression. To confirm this, we expressed either WT MRE11 or a nuclease dead *mre11*^*H129N*^ mutant in isogenic lymphoblastoid TK6 cell lines, and analyzed S9.6 intensity 3 days after inducing MRE11 knockdown using 4-hydroxytamoxifen (4-OHT) treatment (Fig. 5b and Supplementary Fig. 3E)^56^. This analysis showed that MRE11^−/WT^ or MRE11^−/H129N^ TK6 cells did not show significant differences in S9.6 intensity compared with MRE11^WT/WT^ TK6 cells after 4-OHT treatment or to control TK6 cell lines treated with an ethanol control or with no drug treatment. However, despite incomplete knockdown, MRE11^−/−^ TK6 cells showed a twofold increase in mean S9.6 intensity (Fig. 5b). These observations suggest that a non-nucleolytic function of MRE11 is important for R-loop regulation. To gain more insights into this, we monitored the activity of the MRN nuclease on DNA/DNA versus DNA/RNA hybrids (Fig. 5c). The MRN nuclease activity was very efficient on the DNA/DNA substrates, as ~ 50% of the substrate was degraded at 2.5 nm MRN. In contrast, DNA resection was almost undetectable on RNA/DNA substrates, leading to a 50-fold decrease in products. Higher concentration of MRN was required to obtain limited degradation of DNA/RNA hybrids (Fig. 5c). Although it is difficult to know the in vivo concentrations of MRN, especially where it concentrates at DNA damage sites, these studies suggest that MRN nuclease activity is not efficient on DNA/RNA, supporting the idea that this activity does not play a major role in R-loop degradation.

### MRN functions in FA pathway R-loop resolution

**Fig. 6:**
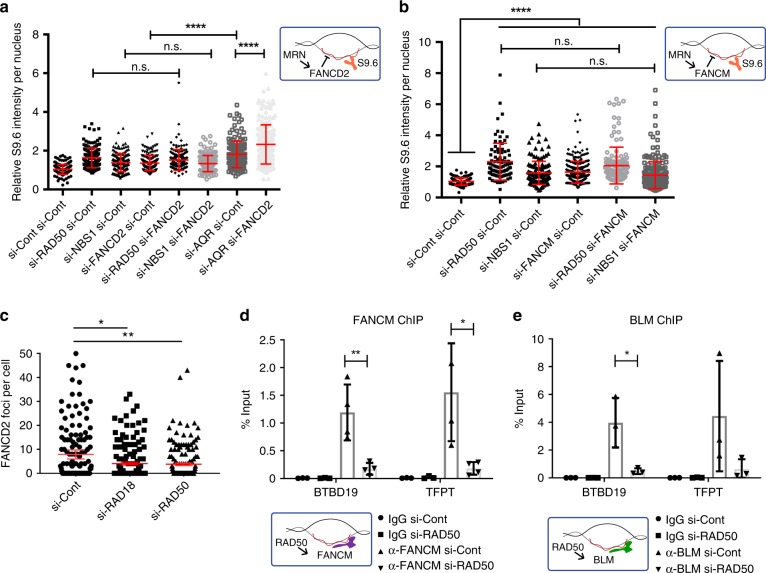
MRN functions in the Fanconi Anemia pathway to recruit FA proteins to R-loops. **a** and **b** Epistatic effects on nuclear S9.6-staining intensity in the indicated conditions. Aquarius (AQR) is a splicing helicase and R-loop regulator in a different pathway, and AQR depletion showed additive nuclear S9.6 staining increases with si-FANCD2 in **a**. *N* = 3; ****P < 0.0001 by ANOVA; mean ± SD. **c** Quantification of nuclear FANCD2 foci by immunofluorescence in the indicated cell conditions. Three replicate experiments were performed and the aggregate foci counts were compared by Fisher Exact test and Bonferroni corrected *p* value cutoffs were used. *P < 0.05 and **P < 0.01. **d** and **e** ChIP of FANCM and BLM at R-loop prone loci BTBD and TFPT depends on RAD50. *N* = 3; *P < 0.05 and **P < 0.01 by *t* test; mean ± SEM. Cartoons illustrating the MRN-FA pathway **a**, **b** and the positive effects of RAD50 on FANCM/BLM recruitment are shown beside each panel

The lack of a direct catalytic role suggested that MRN might function to suppress R-loops through the recruitment of other anti-R-loop activities, such as the FA pathway. To determine whether MRN interacts with the FA pathway, we performed an epistasis experiment examining S9.6 staining in cells doubly depleted by siRNA (Fig. 6a, b, Supplementary Fig. 4A and Supplementary 4B). Depletion of MRN, FANCD2, or FANCM resulted in an expected increase in S9.6 staining, but co-depletion of any of these factors did not further enhance S9.6 staining, suggesting that they work within the same pathway. To confirm that the assay was sufficiently sensitive to observe synergy between separate anti-R-loop pathways, we co-depleted the splicing helicase Aquarius and FANCD2, and observed an additive increase in R-loop levels (Fig. 6a). This supports previous observations linking MRN to FA pathway activation^53^, but places these effects in the context of R-loop tolerance for the first time. To further determine whether MRN functions upstream of FA pathway activation we measured the frequency of FANCD2 nuclear foci in cells depleted for RAD50, or as a control, RAD18, which has previously been implicated in FA activation^57^. FANCD2 foci numbers were significantly reduced in RAD50-depleted cells relative to controls (Fig. 6c). This is consistent with previous data linking MRN to FANCD2 recruitment^53^. Thus, despite perturbed replication and excess DNA damage in RAD50-depleted cells (Figs 2 and 3), FANCD2 recruitment to foci is impaired suggesting a role for MRN upstream of FA.

It has been suggested that the helicase FANCM is a direct effector of R-loops and has the ability to unwind R-loops in vitro^13^. To understand how the MRN complex might impact FANCM recruitment to R-loops, we used ChIP-qPCR to show FANCM binding to R-loop prone loci and found that this interaction was abolished when RAD50 is depleted (Fig. 6d). This indicates that FANCM recruitment to R-loop sites is significantly less efficient in cells lacking MRN, consistent with the observed reduction in FANCD2 foci. It has also been suggested that FANCM acts as an anchor and is required for BLM nuclear localization after replication is stalled^58^. Given that BLM is another helicase, which we previously demonstrated to impact R-loop accumulation^14^, we next determined that BLM binding to R-loop prone loci requires RAD50 (Fig. 6e). Although in vitro treatment of DRIP samples with RNaseH1 clearly reduced DNA:RNA hybrid signal (Fig. 2), the effect of GFP-RNaseH1 expression in cells varied depending on the locus (BTBD19 signal was reduced but TFPT was not; Supplementary Fig. 2E). Indeed, R-loops have been reported to exhibit differing accessibility to RNaseH1 nuclease activity in yeast cells^25^. Thus, it remains unclear whether the RNA moiety is absolutely required for regulating FANCM/BLM recruitment or whether other features of these genes are important. Regardless, these findings support our previous observation that FANCM and BLM have epistatic effects on S9.6 nuclear staining and work in the same R-loop tolerance pathway^14^. Thus, key anti-R-loop proteins within the FA pathway are not recruited effectively to R-loop prone sites in MRN-depleted cells.

## Discussion

**Fig. 7:**
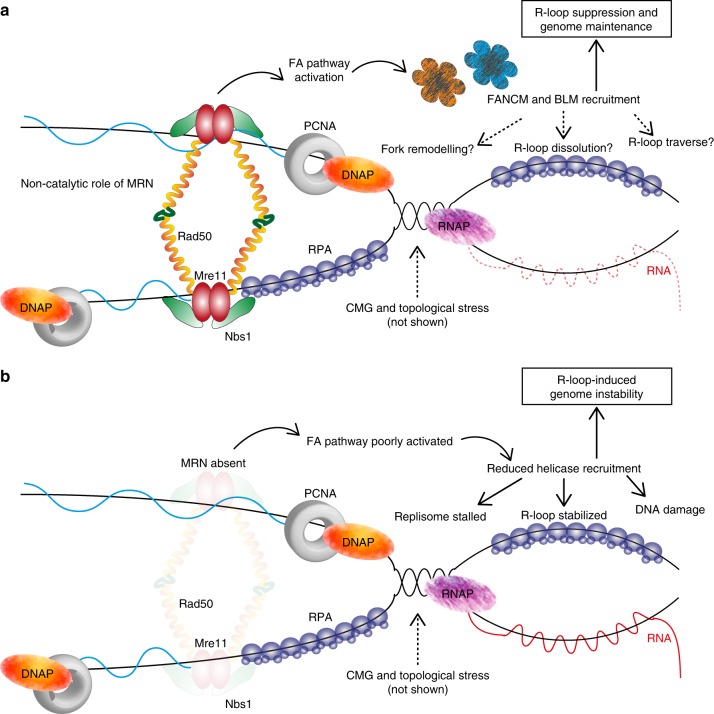
Model of MRN function in R-loop tolerance and mitigation. **a** The MRN complex may play a structural role at transcription–replication conflicts upstream of FA pathway activation and genome stability maintenance. Speculative models for how the FA pathway could resolve these conflicts are indicated with question marks. **b** When MRN is depleted, the FA proteins are poorly recruited to transcription–replication conflicts, R-loops are stabilized, DNA is damaged and genome instability results

Ectopic R-loop accumulation can constitute an endogenous source of genotoxic stress that cells must address to mitigate DNA damage and mutagenesis. Here, we use a cross-species approach and identify the MRN complex as an upstream regulator of R-loop-associated DNA damage. Our data in yeast show that loss of the MRN complex through *RAD50* deletion alters the genomic landscape of R-loops, increasing their abundance at long protein coding genes. We suggest that MRN has opposing roles in R-loop regulation at DNA DSBs compared with replication–transcription conflicts. Our data and the literature support a role for MRN in promoting R-loops to regulate resection at DSBs^30,43^. How MRN might promote transient R-loops at specific types of DNA strand breaks is not clear. In fact CtIP, a factor which stimulates MRN nuclease activity, seems to suppress R-loop formation at sites of radiation damage^59^. What is consistent is that the MRN nuclease activity is likely required to regulate R-loops at induced double-strand breaks^30,59^. In contrast, our data suggest that in the face of aberrant transcription–replication conflicts and stalled replication forks, a structural role for MRN is critical to coordinate R-loop suppression. One mechanism of this regulation is that the recruitment of leading candidates for R-loop removal, FANCM and BLM, to R-loop sites requires MRN. This R-loop-mitigating activity of MRN is independent of its nuclease function and may rely on its reported Structural Maintenance of Chromatin (SMC)-like function in tethering DNA ends^21^. This is consistent with literature suggesting that the yeast MRX complex is important for replisome stability at sites where aberrant transcription has arrested forks in Senataxin-deficient cells^31^. Our data also coalesce with the literature in a model where MRN is an early actor at R-loop stalled forks, and coordinates the recruitment of FA pathway components, destabilizing R-loops, promoting replisome progress, and maintaining genome stability (Fig. 7)^53,59^. These observations also suggest that endogenous transcription–replication conflicts will be important to understanding the roles of MRN in replication in unperturbed cells.

Over the last few decades, R-loops have been linked to transcriptional pausing^60^, termination^61^, splicing^62^, mRNA export,^63^ and other transcription–related phenomena. In this work, we further connect the other component of transcription–replication conflicts, DNA replication, to R-loop tolerance and mitigation. Much of the literature suggests that proteins functioning in the FA pathway at replication forks, including BRCA1, BRCA2, BLM, and XPF, are important for R-loop tolerance and removal^2,10,12–14,60,64^. Our data identify important upstream components to this pathway and raises questions about the unexpected similarities between interstrand cross-link and R-loop bypass or repair. Moreover, other fork-associated proteins implicated in mitigating transcription–replication conflicts, such as WRN, RECQL5, p53, and MCM2, are not as closely linked to the FA pathway^46,65–68^. This may suggest that there are alternative responses to transcription–replication conflicts that depend on the nature of the R-loop itself or the context of the specific DNA sequence (i.e., replication timing, chromatin state). Together, these data also suggest that transcription–replication conflicts caused by R-loops are largely handled by the same machinery that deals with chemical DNA damage. What differentiates transcription–replication conflicts from other damage remains unclear and there is much to learn specifically about how pathway choice downstream of fork stalling is regulated at transcription–replication conflicts.

Finally, it is notable that MRN associates with several helicases that may help to unwind R-loops and facilitate replication fork restart. These helicases include FANCJ which binds directly to MRN^69^, WRN that contacts the NBS1 subunit of MRN^52^, RECQL5, which requires MRN to be recruited to DNA damage sites^70^, and BLM, which exhibits interdependence with MRN recruitment^51,71^. It remains to be determined how these helicases may cooperate or provide specificity to replisome-R-loop conflicts. Moreover, the role and regulation of other anti-R-loop mechanisms (e.g., Senataxin^72^, XPF^10^) in MRN-deficient cells is unclear. Indeed, it is not known why DNA repair helicases are required when both the replisome and transcriptional apparatus are already associated with multiple helicases capable of unwinding R-loops (e.g., MCM at the replisome, Senataxin, RECQL5, DDX19, DDX21 at RNA polymerase)^61,65,68,73,74^. These activities must collaborate or compete to remove the R-loop and restart the replication fork in a timely manner. Another intriguing possibility is that some of these helicases could facilitate R-loop traverse (Fig. 7), where the R-loop is left intact and replication is reinitiated downstream, similar to the role of FANCM and BLM in replisome traverse of interstrand cross-links^75^. As we understand more about R-loop tolerance at endogenous transcription–replication conflicts, the opportunity to probe the coordination of these events with canonical fork restart mechanisms emerges.

## Methods

### Yeast genetic interaction screening and validation

Genetic interactions were identified in yeast using synthetic genetic array^24^. A strain was constructed in the SGA query strain Y7092 with *rnh1*Δ*::NatMX* and *rnh201*Δ*::URA3* deletions and used for mating and selection of double mutant arrays. Colony size was collected on a flatbed scanner and analyzed using the Balony software suite^76^. Candidate interactions were recreated by tetrad dissection. Viable double mutants were analyzed by quantitative growth curves using a Tecan M200 plate reader^77^. In brief, the area under the curve for each growth curve was calculated and fitness values relative to WT were computed for each triplicate measurement. The relative fitness values of the *rnh*ΔΔ strain and a candidate interaction (e.g., *rad50*Δ, *xrs2*Δ) were used to calculate triplicate expected values by multiplication. These expected values were compared to the observed triple mutant fitness values using a *t* test to determine whether a significant genetic interaction occurred. Gene ontology terms were extracted from the Princeton Generic GO term finder (http://go.princeton.edu/cgi-bin/GOTermFinder). Fold enrichments were calculated by computing the ratio of the number of genes associated with GO term in the hitlist and the number of genes associated with the GO term in the genome. Enriched terms and fold enrichment values were plotted using the web-based ReviGO^78^.

### DRIP-chip in yeast

DRIP-chip samples were generated from overnight cultures diluted and grown to an OD600 of 0.7 before cross-linking with 1% formaldehyde for 20 min. Chromatin was purified and sonciated to yield ~ 500 bp fragments, which were incubated with 40 μg of S9.6 antibody (Kerafast Cat #ENH001) and protein A coupled magnetic beads (Invitrogen)^32^. Precipitated DNA was amplified by a double T7 RNA polymerase protocol^79^, biotinylated and hybridized to Affymetrix 1.0 R *S. cerevisiae* microarrays. Samples were normalized to a mock precipitated control using rMAT and relative occupancies were calculated for all probes in a 300 bp sliding window. Replicates were quantile normalized and averaged prior to analysis^14,32^. Enriched features had >1.5-fold enrichment for at least 50% of the probes contained in the feature. Complete data sets can be found at ArrayExpress: E-MTAB-7885. Plots of relative DRIP occupancy over yeast genes were produced by CHROMATRA^80^.

### Yeast chromosome spreads

The indicated mutant strains were grown in rich media and prepared for chromosome spreads and S9.6 staining exactly as described^27^. In brief, spreads were probed with a 1:1000 dilution of S9.6 antibody (Kerafast #ENH001), followed by washing in TBS-T and probing with Cy3-labeled anti-mouse antibody. The proportion of nuclei with visible S9.6 staining was scored and the categorical data was tested using a Fisher Exact test then *p* values were subjected to a Holm–Bonferroni correction to account for multiple comparisons. To determine the variability in WT, 18 replicates were scored and plotted and the screen was completed in duplicate. To verify the signal increase in *rad50*Δ cells is owing to hybrids, chromosome spreads were repeated in triplicate in cells carrying an empty vector, or a plasmid overexpressing yeast Rnh1^14,27^. For this analysis, the fluorescence intensity of individual stained nuclei was measured and pooled data from >250 cells per condition are plotted.

### Cell culture and transfection

HeLa cells were cultivated in Dulbecco’s modified Eagle’s medium (DMEM) (Stemcell technologies) supplemented with 10% fetal bovine serum (Life Technologies) in 5% CO_2_ at 37 °C. The control and inducible CRISPR/Cas9 RNaseH2A knockout HeLa cell lines (gifts from Iain Cheeseman lab)^39^ were maintained in DMEM in the absence of doxycycline. To generate stable clones, Cas9 was induced with 1 μg/ml doxycycline (Sigma) for 3 days before single-cell sorting. Cells were then expanded in the absence of doxycycline and clones with effective RNaseH2A knockout was screened by western blotting. The TK6-derived lymphoblasts were gifts from Hiroyuki Sasanuma and were generated and cultured as previously described^56^. In brief, TK6 cells were grown in RPMI-1640 medium (Stemcell technologies) supplemented with 5% heat-inactivated horse serum and 200 mg/ml sodium pyruvate (Life Technologies). The TK6-derived lymphoblasts were treated with either ethanol (control) or a final concentration of 200 nm 4-hydroxytamoxifen (4-OHT) (H7904, Sigma) for 3 days in the culture medium to generate the MRE11^−/−^ from MRE11^loxp/loxp^, MRE11^−/WT^ from MRE11^loxp/WT^, and MRE11^−/H129N^ from MRE11^loxp/H129N^.

For RNA interference, cells were transfected with siGENOME-SMARTpool siRNAs from Dharmacon (Non-targeting siRNA Pool #1 as si-Cont, si-RAD50, si-NBS1, si-MRE11, si-FANCD2, si-FANCM, si-AQR, si-RAD18). Transfections were done with Dharmafect1 transfection reagent (Dharmacon) according to manufacturer’s protocol and harvested 48 h after the siRNA administration. For experiments with overexpression of GFP or nuclear-targeting GFP-RNaseH1 (gift from R. Crouch), transfections were performed with Lipofectamine 3000 (Invitrogen) according to manufacturer’s instructions 24 h after the siRNA transfections.

### Western blotting

Whole-cell lysates were prepared with RIPA buffer containing protease inhibitor (Sigma) and phosphatase inhibitor (Roche Applied Science) cocktail tablets and the protein concentration were determined by Bio-Rad Protein assay (Bio-Rad). Equivalent amounts of protein were resolved by SDS-PAGE and transferred to polyvinylidene fluoride microporous membrane (Millipore), blocked with 5% skim milk in TBS containing 0.1% Tween-20 (TBS-T), and membranes were probed with the following antibodies: RAD50 (1:1000, [13B3/2C6]ab89, abcam), γH2AX (1:5000, [EP854(2)Y]ab81299 abcam), FANCM (1:1000, ab95014,abcam), NBS1 (1:1000, A300-187A-T, Bethyl laboratories), MRE11 (1:1000, A300-181A-T, Bethyl laboratories), RNaseH2A (1:1000, A304-149A, Bethyl laboratories), AQR (1:1000, A302-547A, Bethyl laboratories), H2AX (1:500, [D17A3]XP #7631, cell signaling), p-ATM (1:500, (10H11.E12) sc-47739, Santa Cruz), p-CHK2 (Th468) (1:1000, C13C1, cell signaling), p-ATR (Ser428) (1:1000, #2853, cell signaling), p-Chk1 (Ser345) (1:1000, 133D3, cell signaling), FANCD2 (1:1000, NB100-182SS, Novus), GFP Tag (GF28R) (1:4000, MA5-15256, ThermoFisher Scientific), GAPDH (GA1R) (1:3000, MA5-15738, ThermoFisher Scientific), and α-tubulin (1:3000, B-5-1–2, ThermoFisher Scientific). Secondary antibodies were conjugated to horseradish peroxidase (HRP) and peroxidase activity was visualized using Chemiluminescent HRP substrate (Thermo Scientific). Western blot densitometry analysis was performed using ImageJ and are expressed relative to the first lane control and normalized to the indicated loading control.

### Crystal violet cell viability assay

Control and RNaseH2A CR-knockout cells were counted and seeded in 12-well plates. Forty-eight hours post siRNA treatment, cells were washed with PBS, fixed with 10% formalin for 30 min, and stained with 0.05% crystal violet for 30 min at room temperature. The relative levels of stain intensity were quantified by washing the stain with 10% acetic acid in methanol and measuring the absorbance at 590 nm using a TECAN M200. The relative absorbance at 590 nm was used to compute expected and observed values based on a multiplicative model of fitness^81^.

### Immunofluorescence

For S9.6 staining, cells were grown on coverslips overnight before siRNA transfection and plasmid overexpression. Forty-eight hours post siRNA transfection, cells were washed with PBS, fixed with ice-cold methanol for 10 min and permeabilized with ice-cold acetone for 1 min. After PBS wash, cells were blocked in 3% bovine serum albumin (BSA), 0.1% Tween-20 in 4× saline sodium citrate buffer (SSC) for 1 h at room temperature. Cells were then incubated with primary antibody S9.6 (1:500, S9.6, Kerafast) overnight at 4 °C. Cells were then washed three times in PBS and stained with mouse Alexa-Fluor-488 or 568-conjugated secondary antibody (1:1000, Life Technologies) for 1 h at room temperature, washed three times in PBS, and stained with DAPI for 5 min. Cells were imaged on LeicaDMI8 microscope at ×100 and ImageJ was used for processing and quantifying nuclear S9.6 intensity in images. For experiments with GFP overexpression, only GFP-positive cells were quantified. For cells with in vitro recombinant RNaseH or RNaseIII treatment, cells were treated with recombinant RNaseH overnight or ShortCut RNaseIII (New England Biolabs) for 20 min at 37 °C before proceeding with blocking. For γH2AX, FANCD2, and p-ATM foci, the immunostaining was performed the same way except fixation with 4% paraformaldehyde for 15 min and permeabilization with 0.2% Triton X-100 for 5 min on ice for γH2AX (1:1000, H2A.X(phosphor S139) [EP854(2)Y], ab81299, abcam), FANCD2 (1:1000, NB100-182SS, Novus) and p-ATM (1:500, [10H11.E12], sc-47739, Santa Cruz). Secondary antibody was rabbit Alexa-Fluoro-488 or 568-conjugated antibody (1:1000) (Life Technologies).

### Comet assay

The neutral comet assay was performed using the CometAssay Reagent Kit for Single Cell Gel Electrohoresis Assay (Trevigen) in accordance with the manufacturer’s instructions. In brief, cells were combined with LMAgarose at 37 °C at a ratio of 1:10 (cell:agarose) and spread onto CometSlide. After gelling in 4 °C in the dark, the slides were then immersed in 4 °C Lysis Solution overnight. The next day, slides were removed from Lysis Solution and immersed in 4 °C 1 × Neutral Electrophoresis Buffer for 30 min. Electrophoresis was then performed at 4 °C at 20 Volts for 30 min. Slides were then immersed in DNA Precipitation Solution for 30 min followed by 70% ethanol for 30 min at room temperature, and dried at 37 °C for 10 min. Finally, slides were stained with PI and imaged on LeicaDMI8 microscope at ×20. Comet tail moments were obtained using an ImageJ plugin as previously described^82^. At least 50 cells per sample were analyzed from each independent experiment.

### Native BrdU

Cells were grown on coverslips overnight before siRNA transfection and plasmid overexpression. Forty-eight hours post siRNA transfection, a final concentration of 30 μm BrdU (Sigma) was added to the culture medium and incubated for 24 h at 37 °C. After a 24 h incubation with BrdU, cells were rinsed with PBS twice, fixed with ice-cold 70% ethanol for 10 min and blocked with 3% BSA, 0.1% Tween-20 in 4 × SSC for 1 h at room temperature. Cells were than incubated with anti-BrdU antibody (1:500, 555627, BD) for 1 h at room temperature, washed three times in PBS and stained with mouse Alexa-Fluoro-568-conjugated secondary antibody (1:1000) (Life Technologies) for 1 h at room temperature. Cells were then stained with DAPI for 5 min and imaged on LeicaDMI8 microscope at ×100. ImageJ was used for processing and quantifying nuclear BrdU intensity in images.

### X-ray irradiation

Irradiation was carried out with a Precision X-Ray X-RAD320 Series X-ray machine. For S9.6 and γH2AX immunofluorescence, HeLa cells were irradiated at a dose of 1 Gy for 11 s, 300 kV, 10 mA. For ATM phosphorylation on Ser1981, U2OS cells were first synchronized in G1/S using double thymidine block, treated with 50 μm Mirin for 30 min and then irradiated at a dose of 10 Gy, as described^55^. Immediately after irradiation, cells were put back into the 37 °C incubator for 30 min before fixing with either methonal (for S9.6) or 4%PFA (for γH2AX and p-ATM) for immunofluorescence analysis as above.

### Proximity ligation assay

Cells were grown on coverslips, washed with PBS, fixed with 4% paraformaldehyde for 15 min and permeabilized with 0.2% Triton X-100 for 5 min. Cells were then blocked in 3% BSA, 0.1% Tween-20 in 4XSSC for 1 h at room temperature. Cells were then incubated with primary antibody overnight at 4 °C [1:500 goat anti-RNA polymerase II antibody (PLA0292, Sigma) with 1:500 rabbit anti-PCNA antibody (PLA0079, Sigma)]. The next day after washing with 1 ×  PBS twice, cells were incubated with pre-mixed PLA probe anti-goat minus and PLA probe anti-rabbit plus (Sigma) for 1 h at 37 °C. The subsequent steps in proximal ligation assay were carried out with Duolink In Situ Kit (Sigma) in accordance to manufacturer’s instructions. In brief, cells were washed with Buffer A for 5 min three times, ligation reaction for 30 min at 37 °C, washed with Bufer A for 2 min two times, and Amplification for 100 min at 37 °C. After washing with Buffer B for 10 min three times and 0.01% Buffer B for 1 min, slides were then stained with DAPI and imaged on LeicaDM18 microscope at ×100. Negative controls were treated identically but anti-RNA polymerase II antibody was omitted.

### Genomic DNA extraction and DNA combing

Cells were first pulse labeled for 20 min with 250 μm IdU (Sigma), washed twice with PBS, and then pulse labeled with 30 μm CldU (Sigma) for 20 min. Cells were then collected and scraped into ice-cold PBS and genomic DNA was extracted with CombHeliX DNA Extraction kit (Genomic vision) in accordance with the manufacturer’s instructions. DNA fibers were stretched on vinyl silane-treated glass coverslips (CombiCoverslips) (Genomic vision) with automated Molecular Combing System (Genomic Vision). After Combing, the stretched DNA fibers were dehydrated in 37 °C for 2 h, fixed with MeOH:Acetic acid (3:1) for 10 min, denatured with 2.5 m HCl for 1 h, and blocked with 5% BSA in PBST for 30 min. IdU and CldU were then detected with the following primary antibodies in blocking solution for 1 h at room temperature: mouse anti-BrdU (B44) (1:40) (BD) for IdU and rat anti-BrdU [Bu1/75 (ICR1)] (1:50) (abcam) for CldU. After PBS wash, fibers were than incubated with secondary antibodies anti-Rat-Alexa488 (1:50) (Invitrogen) and anti-mouse Alexa 568 (1:50) (Life Technologies) for 1 h at room temperature. DNA fibers were analyzed on LeicaDM18 microscope at ×100 and ImageJ was used to measure fiber length. For the experiment with transcription inhibitor, cells were first treated with DMSO or 50 μm cordycepin for 2 h before IdU labeling.

### SIRF

SIRF (in situ protein interactions at nascent and stalled replication forks) was carried out as previously described^47^. HeLa cells were seeded on coverslips and grown overnight before transfecting with GFP or GFP-RNH1 plasmids. The next day, cells were incubated with 125 μm EdU for 8 min. After EdU was removed and cells were washed with PBS for two times, fresh growth media or media containing 5 μm pladienolide B (PlaB) (Santa cruz) or 2 mm HU (Sigma) were added for 2 h. After 2 h of drug treatment, media was removed and cells were washed with PBS two times before fixation with 3% PFA and permiabilized with 0.25% TritonX-100 in PBS. After blocking, cells were incubated with the following primary antibodies overnight: mouse anti-Mre11 [12D7] ab214 (1:200) (abcam) and rabbit anti-biotin (D5A7) (1:200) (Cell signaling). The rest of the protocol follows Proximal Ligation Assay as mentioned above.

### FACS analysis for S9.6

Cells were harvested, washed with 1 × PBS twice, extracted with 25 mm HEPES (pH 7.4), 50 mm NaCl, 1 mm EDTA with protease inhibitor on ice, and fixed with 2% paraformaldehyde in 1 × PBS^83^. Cells were then incubated with primary antibody S9.6 (1:200, clone S9.6, MABE1095, millipore) overnight at 4 °C and with anti-mouse-IgG-PerCP-Cy5.5 secondary antibody (1:100) (Santa Cruz) for 30 min at room temperature. Cells were resuspended in 1 × PBS and analyzed with a FACSCalibur flow cytometer using CellQuestPro software. The mean S9.6 intensity and histograms were generated using FlowJo Version 9.3.2.

### DRIP and ChIP-qPCR

The method was followed from ref. ^84^ with some modifications. Cells were cross-linked in 1% formaldehyde for 10 min before quenching with glycine for 5 min at room temperature, and then lysed in ChIP lysis buffer (50 mm HEPES-KOH at pH 7.5, 140 mm NaCl, 1 mm EDTA at pH 8, 1% Triton X-100, 0.1% Na-Deoxycholate, 1% SDS) and rotated for 1 h at 4 °C. DNA were sonicated on Q Sonica Sonicator Q700 for 8 min (30 sec ON, 30 s OFF) to generate fragments of 200–500 bp. For DRIP, the chromatin preps were treated with 20 mg/mL Proteinase K (Thermo Fisher Scientific) at 65 °C overnight and total DNA was purified by phenol/chloroform purification method. Protein A magnetic beads (Bio-rad) were first pre-blocked with PBS/EDTA containing 0.5% BSA and then incubated with S9.6 antibody (1:200, clone S9.6, MABE1095, Millipore) in IP buffer (50 mm Hepes/KOH at pH 7.5; 0.14 m NaCl; 5 mm EDTA; 1% Triton X-100; 0.1% Na-Deoxycholate, ddH_2_O) at 4 °C for 4 h with rotation. DNA was then added to the mixture and gently rotated at 4 °C overnight. Beads were recovered and washed successively with low salt buffer (50 mm Hepes/KOH pH 7.5, 0.14 m NaCl, 5 mm EDTA pH 8, 1% Triton X-100, 0.1% Na-Deoxycholate), high salt buffer (50 mm Hepes/KOH pH 7.5, 0.5 m NaCl, 5 mm EDTA pH 8, 1% Triton X-100, 0.1% Na-Deoxycholate), wash buffer (10 mm Tris-HCl pH 8, 0.25 m LiCl, 0.5% NP-40, 0.5% Na-Deoxycholate, 1 mm EDTA pH 8), and TE buffer (100 mm Tris-HCl pH 8, 10 mm EDTA pH 8) at 4 °C, two times. Elution was performed with elution buffer (50 mm Tris-HCl pH 8, 10 mm EDTA, 1% SDS) for 15 min at 65 °C. After purification with PCR Cleanup kit (Sigma-Aldrich), nucleic acids were eluted in 100 μL of elution buffer (5 mm Tris-HCl pH 8.5) and analyzed by quantitative real-time PCR (qPCR). For ChIP, DNA and antibody were incubated in IP buffer overnight with rolling at 4 °C. Antibodies used were FANCM (1:150, ab95014, abcam), BLM (1:150, ab2179, abcam), MRE11 (1:150, ab33125, abcam). Antibody-bound DNA was recovered using the Protein A or Protein G magnetic beads (Bio-Rad), washed similarly as DRIP samples and treated with Proteinase K and RNAse after elution. Then, antibody-bound DNA was purified with PCR Cleanup kit and analyzed by qPCR. qPCR was performed with Fast SYBR Green Master (ABI) on AB Step One Plus real-time PCR machine (Applied Biosystem). Primer sequences are BTBD19-forward (5′-CCCCAAAGGGTGGTGACTT-3′), BTBD19-reverse (5′-TTCACATTACCCAGACCAGACTGT-3′), TFPT-forward (5′-TCTGGGAGTCCAAGCAGACT-3′) and TFPT-reverse (5′-AAGGAGCCACTGAAGGGTTT-3′). qPCR results were analyzed using the comparative CT method. The RNA–DNA hybrid and ChIP DNA enrichments were calculated based on the IP/Input ratio.

### MRN nuclease assays

The substrates used were DNA/DNA hybrid (JYM3395 GTTTCTGGACCATATGATACATGCTCTGGCCAAGCATTCCGGCTGGTCGCTAATCGTTGA and JYM3393 TCAACGATTAGCGACCAGCCGGAATGCTTGGCCAGAGCATGTATCATATGGTCCAGAAAC) or DNA/RNA hybrid (JYM3395 GTTTCTGGACCATATGATACATGCTCTGGCCAAGCATTCCGGCTGGTCGCTAATCGTTGA and JYM3394 rUrCrArArCrGrArUrUrArGrCrGrArCrCrArGrCrCrGrGrArArUrGrCrUrUrGrGrCrCrArGrArGrCrArUrGrUrArUrCrArUrArUrGrGrUrCrCrArGrArArArC). JYM3395 was end-labeled in 5′ with γ-ATP and T4 polynucleotide kinase (NEB) according to the manufacturer’s conditions. The complementary strand was annealed and the DNA/DNA or DNA/RNA hybrids were purified from an 8% PAGE gel.

MRN exonuclease reactions were performed in 25 mm MOPS (morpholinepropanesulfonic acid; pH 7.0), 60 mm KCl, 0.2% Tween-20, 2 mm dithiothreitol, 2 mm ATP and 5 mm MnCl_2_, with 100 nm DNA/DNA hybrid or DNA/RNA hybrid radiolabeled substrates. Reaction mixtures were incubated for 1 h at 37 °C, followed by treatment with one-fifth volume of stop buffer (20 mm Tris–Cl pH 7.5 and 2 mg/mL proteinase K) for 30 min at 37 °C. Formamide loading buffer was added to the reaction mixtures, which were heated at 95 °C for 5 min and then loaded onto 10% polyacrylamide denaturing gels. Gels were migrated at 55 W for 120 min and then exposed in a phosphorimager cassette or on film. The percentages of DNA resection, from three independent experiments, was calculated from the intact DNA substrate in the reaction mixtures divided by the intact DNA substrate in the no enzyme control. This provided the percentage of DNA remaining after resection, which was substracted from 100.

### Reporting summary

Further information on research design is available in the Nature Research Reporting Summary linked to this article.

## Supplementary information

Supplementary Information

Description of Additional Supplementary Files

Supplementary Data 1

Reporting Summary

Source Data

## Data Availability

Complete data sets for DRIP-chip can be found at ArrayExpress with the accession number E-MTAB-7885. All other data supporting the findings of this study are available within the article and its Supplementary Information files, or from the authors upon reasonable request.
