# Supplementary material for: MRE11-RAD50-NBS1 promotes Fanconi Anemia R-loop suppression at transcription–replication conflicts
Source: Nat Commun. 2019 Sep 19;10:4265. doi: 10.1038/s41467-019-12271-w (PMC6753070; doi:10.1038/s41467-019-12271-w)
Supplement: Supplementary file 1 — Supplementary Information [file 41467_2019_12271_MOESM1_ESM.pdf]

Supplementary Information for:

MRE11-RAD50-NBS1 promotes Fanconi Anemia R-loop suppression at transcription-replication conflicts

Emily Yun-chia Chang[1], Shuhe Tsai[1], Maria J. Aristizabal[2], James P. Wells[1], Yan Coulombe[3,4], Franciele F. Busatto [3,4], Yujia A. Chan[5], Arun Kumar[1], Yi Dan Zhu[1], Alan Ying-Hsu Wang[1], Louis-Alexandre Fournier[1], Philip Hieter[6,7], Michael S. Kobor[2], Jean-Yves Masson[3,4], Peter C. Stirling[1,7]

Supplementary Information List

Supplementary Dataset 1. **List of SGA results for *rnh1*Δ, *rnh201*Δ and *rnh1*Δ*rnh201*Δ (see dataset file).**

Supplementary Figure 1. Yeast *RAD50* regulates DNA:RNA hybrids.

Supplementary Figure 2. Western blots, S9.6 imaging controls, DRIP controls, and comet tail images.

Supplementary Figure 3. **Functional testing of the HU and Mirin concentrations.**

Supplementary Figure 4. Double siRNA knockdown efficiencies.

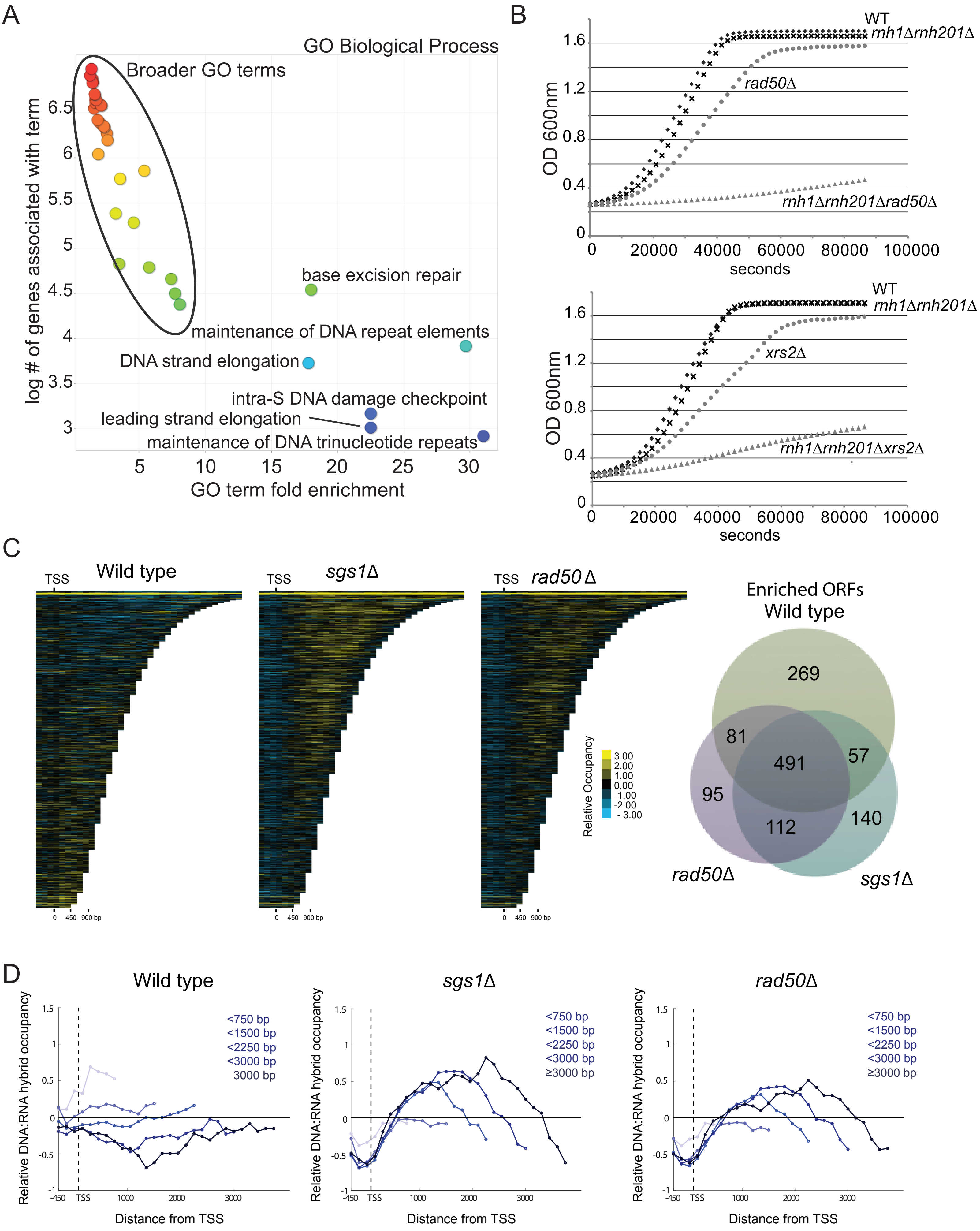

Chang et al., Supplementary Figure 1

Supplementary Figure 1. Yeast *RAD50* regulates DNA:RNA hybrids. (A) Gene Ontology analysis of negative *rnh*ΔΔ interacting partners. Plots show the output of GO Biological Processes by ReviGO. (B) Representative growth curves of *rad50*Δ or *xrs2*Δ with *rnh*ΔΔ. Statistical analysis of area under the curve for triplicates confirm that the triple mutants are significantly sicker than expected, p<0.001. (C) Comparison of DRIP-chip profiles for the indicated strains. Left, Chromatra plots illustrate occupancy of hybrids shifting to longer genes in mutant strains. Right, Venn diagram of genes enriched for DNA::RNA hybrids in the WT, and mutant strains show similarities and differences between the mutant profiles. (D) Mean genome- DNA:RNA hybrid occupancy in WT, *sgs1*Δ or *rad50*Δ as a function of gene length. A total of 4,868 genes were split into the indicated gene length categories (538 genes < 750 bp, 1,861 genes < 1,500 bp, 1,263 genes < 2,250 bp, 636 genes < 3,000 bp, and 570 genes ≤ 3,000 bp) with mean enrichment scores calculated and plotted for each category.

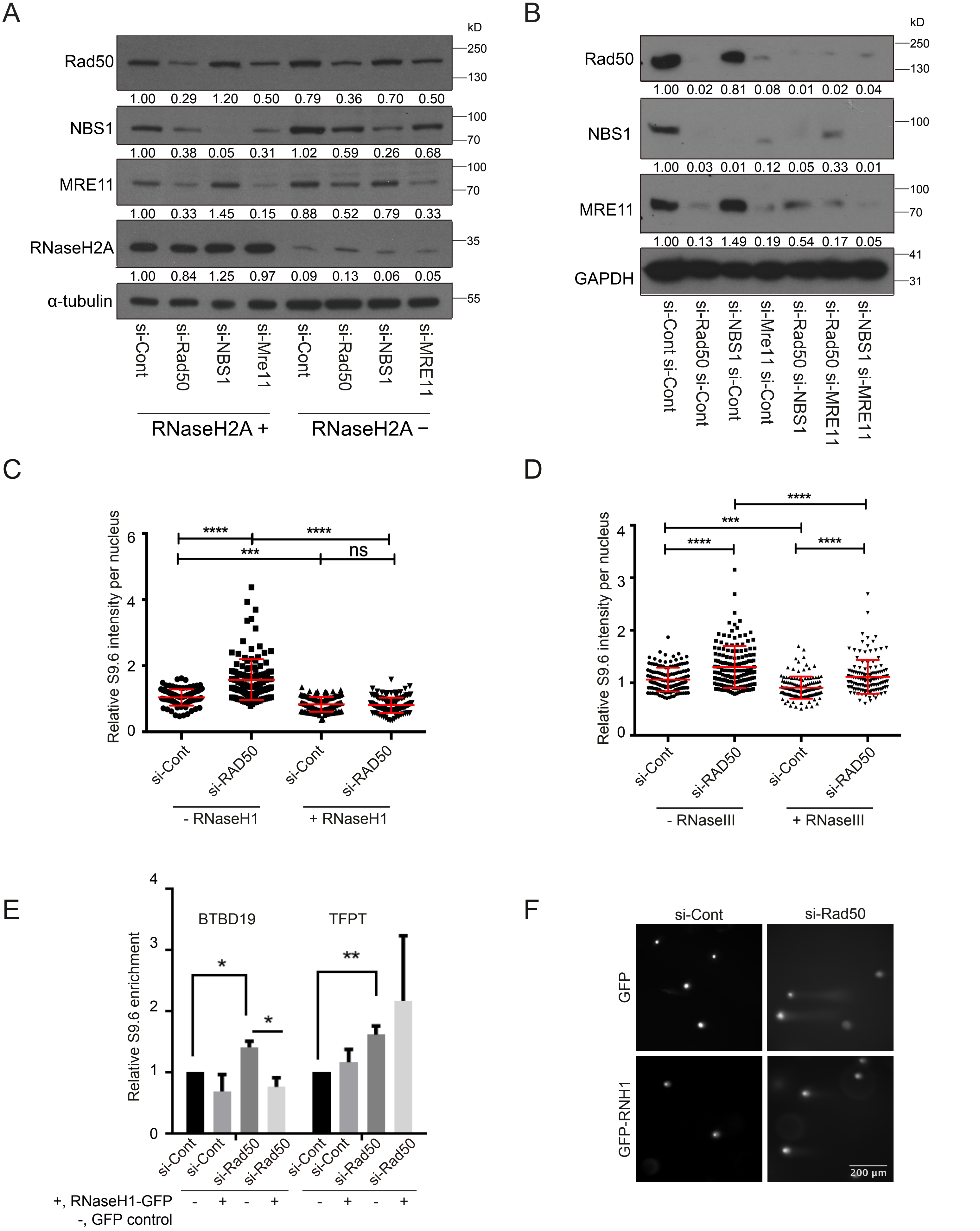

Chang et al., Supplementary Figure 2

Supplementary Figure 2. Western blots, S9.6 imaging controls, DRIP controls, and comet tail images. (A) Western blot showing effective knockdown of the MRN complex in control and dox-induced RNaseH2A deficient HeLa cells. α-tubulin served as loading control. (B) Western blot confirming siRNA knockdown levels of MRN complex from R-loop staining experiments in main text Figure 2B. (C and D) *In vitro* treatment of slides for immunofluorescence of S9.6 in control or RAD50-depleted cells. C shows that RNaseH treatment removes differences in staining intensity between control and si-RAD50. D shows that RNaseIII treatment to remove dsRNA reduces background staining but the difference between control and si-RAD50 persists. ***$P<0.001$ and ****$P < 0.0001$ by ANOVA; mean ± SD. (E) DRIP-qPCR signal at BTBD19 and TFPT when cotransfected with GFP or GFP-RNaseH1. DRIP signal is induced by siRAD50 and this is significantly reduced if cells are cotransfected with GFP-RNaseH1 relative to a GFP control. DRIP signal at TFPT is also induced by RAD50 depletion but is not reduced by co-transfection of GFP-RNaseH1. *In vitro* treatment of chromatin input with RNaseH (main text Figure 2D) shows that both genes have DNA:RNA hybrids that drive the DRIP signal. Why the efficacy of RNaseH1 varies at different loci in cells is unclear. *$P<0.05$ and **$P < 0.01$ by ANOVA; mean ± SD. (F) Representative comet tail images from single-cell electrophoresis in main text Figure 2F.

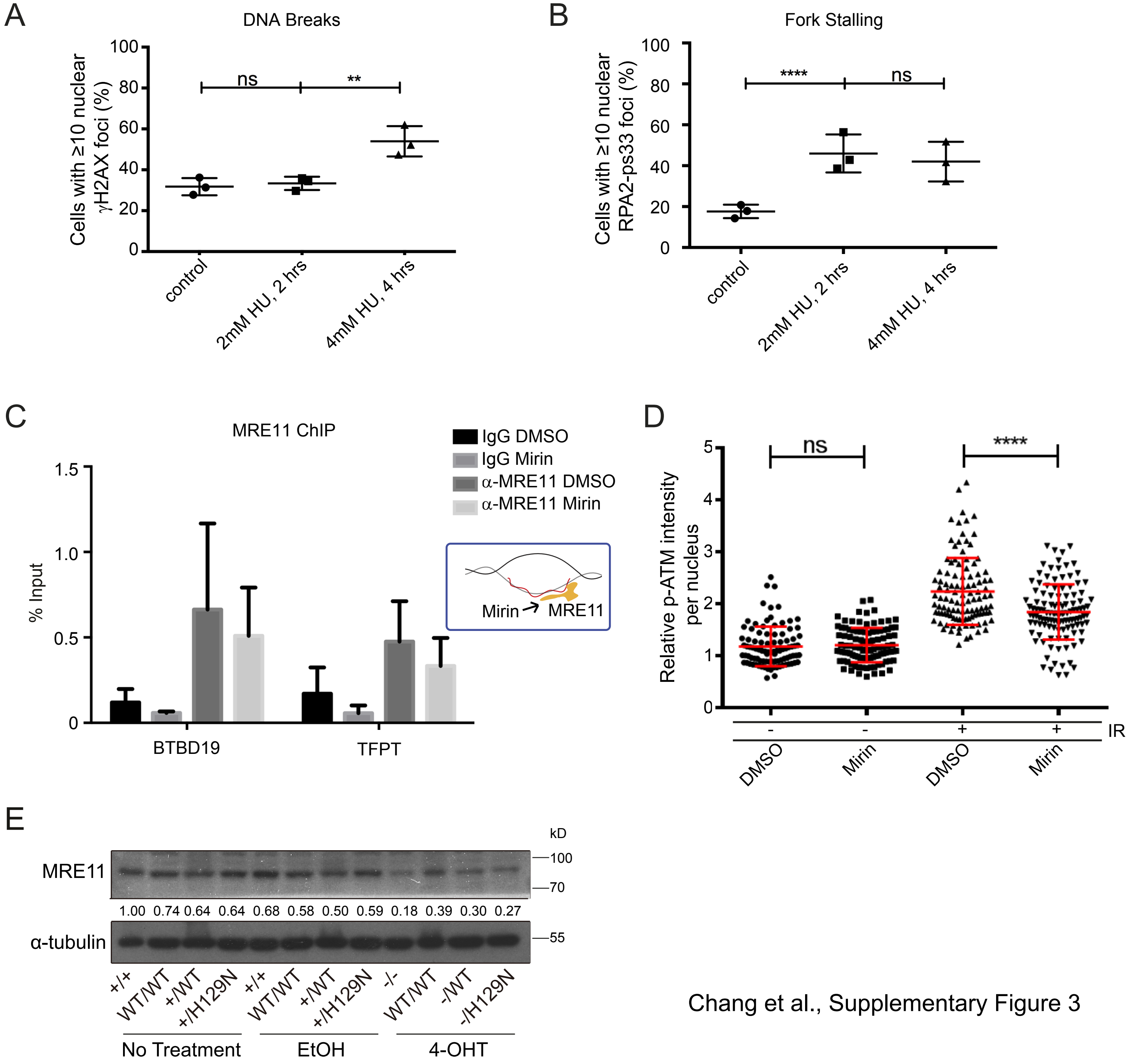

Chang et al., Supplementary Figure 3

Supplementary Figure 3. **Functional testing of the HU and Mirin concentrations.** (A) Scoring of induced γ-H2AX foci after the indicated HU treatment. (B) Scoring of induced RPA2-Ser33-phosphorylation after the indicated HU treatment. A and B, show that both HU concentrations induced replication stress, but only the higher and longer treatment likely elicits DSBs. **P<0.01 and ****P < 0.0001 by Fisher's exact test; mean ± SD. (C) Mre11 ChIP to R-loops prone genes. Treatment with Mirin does not significantly reduce the ChIP of Mre11 to R-loop prone loci BTBD19 or TFPT. (D) Confirmation of Mirin effects on ATM phosphorylation. Mirin stocks are effective at reducing IR induced ATM phosphorylation. As in Dupre, et al., 2008 U2OS cells treated with Mirin do not enact normal ATM phosphorylation after irradiation because of Mre11 inhibition[55]. ****P < 0.0001 by *t* test (E) Western blot showing MRE11 protein levels after 3-day treatment with EtOH (control) or 4-OHT, to induce gene knockout, in the TK6-derived lymphoblasts of the indicated genotype. α-tubulin served as loading control. Related to Figure 5B.

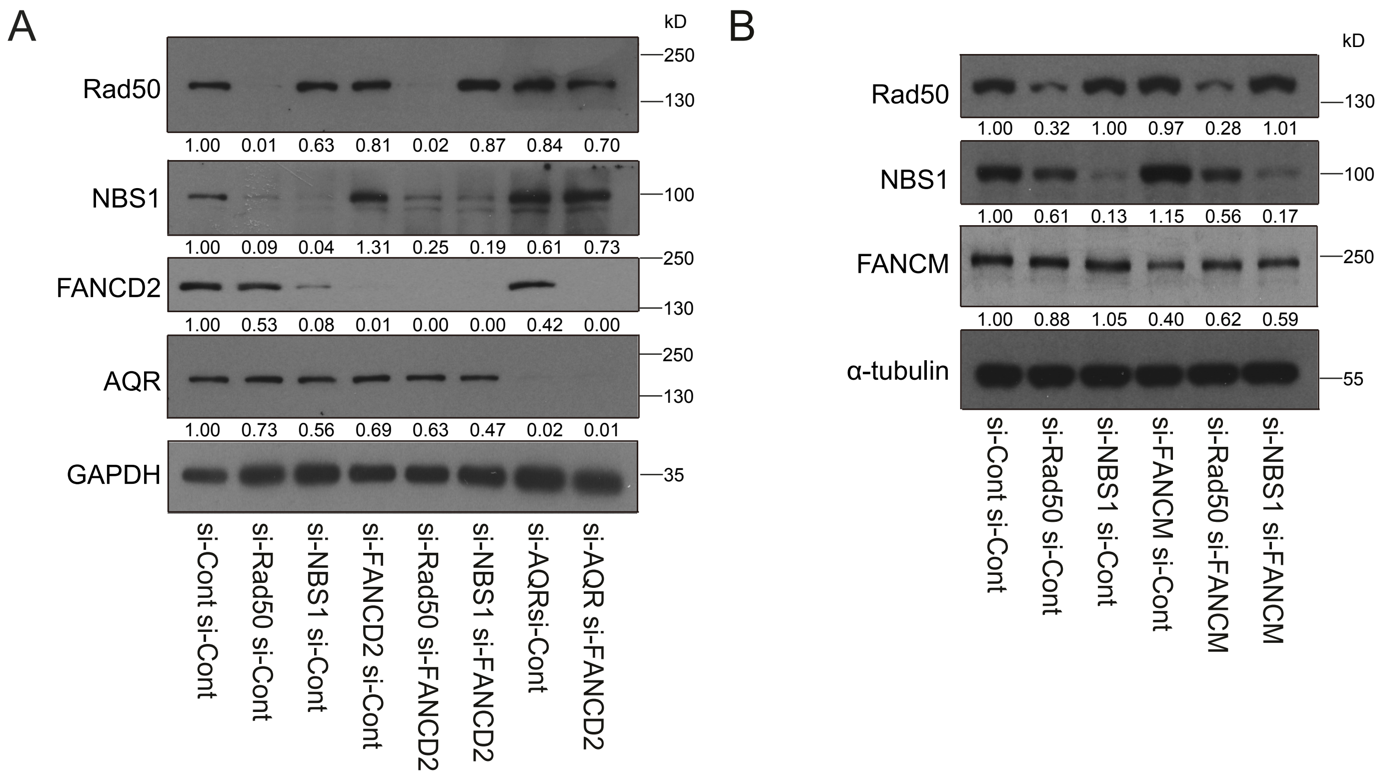

Chang et al., Supplementary Figure 4

Supplementary Figure 4. Double siRNA knockdown efficiencies. (A and B) Western blot confirming double siRNA knockdown levels from R-loop staining experiments in main text Figure 6 A-B.
