# Supplementary material for: MRE11-RAD50-NBS1 promotes Fanconi Anemia R-loop suppression at transcription–replication conflicts
Source: Nat Commun. 2019 Sep 19;10:4265. doi: 10.1038/s41467-019-12271-w (PMC6753070; doi:10.1038/s41467-019-12271-w)
Supplement: Supplementary file 2 — Description of Additional Supplementary Files [file 41467_2019_12271_MOESM2_ESM.pdf]

Description of Additional Supplementary Files

File Name: Supplementary Data 1
Description: Synthetic genetic arrays scores and p-values. For each SGA screen the gene name and allele type as listed along with the experimental-control (E-C) score value and a p-value for hits with E-C <-0.2 and p<0.05.
