# Supplementary material for: MRE11-RAD50-NBS1 promotes Fanconi Anemia R-loop suppression at transcription–replication conflicts
Source: Nat Commun. 2019 Sep 19;10:4265. doi: 10.1038/s41467-019-12271-w (PMC6753070; doi:10.1038/s41467-019-12271-w)
Supplement: Supplementary file 4 — Reporting Summary [file 41467_2019_12271_MOESM4_ESM.pdf]

nature research

Corresponding author(s): Peter Stirling

Last updated by author(s): Jul 29, 2019

Reporting Summary

Nature Research wishes to improve the reproducibility of the work that we publish. This form provides structure for consistency and transparency in reporting. For further information on Nature Research policies, see Authors & Referees and the Editorial Policy Checklist.

Statistics

For all statistical analyses, confirm that the following items are present in the figure legend, table legend, main text, or Methods section.

n/a	Confirmed	
☐	☒	The exact sample size (n) for each experimental group/condition, given as a discrete number and unit of measurement
☐	☒	A statement on whether measurements were taken from distinct samples or whether the same sample was measured repeatedly
☐	☒	The statistical test(s) used AND whether they are one- or two-sided<br>*Only common tests should be described solely by name; describe more complex techniques in the Methods section.*
☒	☐	A description of all covariates tested
☐	☒	A description of any assumptions or corrections, such as tests of normality and adjustment for multiple comparisons
☐	☒	A full description of the statistical parameters including central tendency (e.g. means) or other basic estimates (e.g. regression coefficient) AND variation (e.g. standard deviation) or associated estimates of uncertainty (e.g. confidence intervals)
☐	☒	For null hypothesis testing, the test statistic (e.g. F, t, r) with confidence intervals, effect sizes, degrees of freedom and P value noted<br>*Give P values as exact values whenever suitable.*
☒	☐	For Bayesian analysis, information on the choice of priors and Markov chain Monte Carlo settings
☒	☐	For hierarchical and complex designs, identification of the appropriate level for tests and full reporting of outcomes
☒	☐	Estimates of effect sizes (e.g. Cohen's d, Pearson's r), indicating how they were calculated

Our web collection on statistics for biologists contains articles on many of the points above.

Software and code

Policy information about availability of computer code

Data collection	*Provide a description of all commercial, open source and custom code used to collect the data in this study, specifying the version used OR state that no software was used.*
Data analysis	*Provide a description of all commercial, open source and custom code used to analyse the data in this study, specifying the version used OR state that no software was used.*

For manuscripts utilizing custom algorithms or software that are central to the research but not yet described in published literature, software must be made available to editors/reviewers. We strongly encourage code deposition in a community repository (e.g. GitHub). See the Nature Research guidelines for submitting code & software for further information.

Data

Policy information about availability of data

All manuscripts must include a data availability statement. This statement should provide the following information, where applicable:

- Accession codes, unique identifiers, or web links for publicly available datasets
- A list of figures that have associated raw data
- A description of any restrictions on data availability

DRIP-chip data collection and analysis was performed exactly as described previously14. Complete datasets can be found at ArrayExpress: : E-MTAB-7885.

Field-specific reporting

Please select the one below that is the best fit for your research. If you are not sure, read the appropriate sections before making your selection.

☒ Life sciences ☐ Behavioural & social sciences ☐ Ecological, evolutionary & environmental sciences

Life sciences study design

All studies must disclose on these points even when the disclosure is negative.

Sample size	Sample size was not predetermined.
Data exclusions	No data was excluded
Replication	All experiments were replicated, see the methods section for specifics of each experiment.
Randomization	Samples were not allocated into experimental groups
Blinding	Investigators were not blinded to sample identity.

Reporting for specific materials, systems and methods

We require information from authors about some types of materials, experimental systems and methods used in many studies. Here, indicate whether each material, system or method listed is relevant to your study. If you are not sure if a list item applies to your research, read the appropriate section before selecting a response.

Materials & experimental systems

n/a	Involved in the study	
☐	☒	Antibodies
☐	☒	Eukaryotic cell lines
☒	☐	Palaeontology
☒	☐	Animals and other organisms
☒	☐	Human research participants
☒	☐	Clinical data

Methods

n/a	Involved in the study	
☒	☐	ChIP-seq
☐	☒	Flow cytometry
☒	☐	MRI-based neuroimaging

Antibodies

Antibodies used

Mouse anti-DNA-RNA Hybrid[S9.6] (Kerafast),
Mouse anti-DNA-RNA Hybrid, clone S9.6 (MABE1095) (Millipore),
Mouse anti-Rad50 [13B3/2C6] ab89 (abcam),
Rabbit anti-Mre11 (A300-181A-T) (Bethyl Laboratories),
Rabbit anti-NBS1 (A300-187A-T) (Bethyl Laboratories),
Rabbit anti-RNaseH2A (A304-149A) (Bethyl Laboratories),
Mouse anti-Phospho-ATM (10H11.E12) (sc-47739) (SantaCruz),
Rabbit anti-Phospho-ATR (Ser428) (#2853) (Cell signaling),
Rabbit anti-Phospho-Chk1 (Ser345)(133D3) (Cell signaling),
Rabbit anti-Phospho-Chk2 (Thr68)(C13C1) (Cell signaling),
Rabbit anti-Histone H2A.X(phosphor S139) [EP854(2)Y] (ab81299) (abcam),
Rabbit anti-Histone H2A.X (D17A3) XP (#7631) (Cell signaling),
Rabbit anti-IBP160 (AQR) (A302-547A) (Bethyl Laboratories),
Rabbit anti-FANCD2(NB100-182SS) (Novus),
Rabbit anti-FANCM (ab95014) (abcam),
Rabbit anti-BLM (ab2179) (abcam),
Rabbit anti-Phospho RPA32(S33) (A300-246A) (Bethyl Laboratories),
Mouse anti-BrdU (555627) (BD),
Mouse anti-GAPDH (GA1R) (MA5-15738) (ThermoFisher Scientific),
Mouse anti-GFP Tag (GF28R) (MA5-15256) (ThermoFisher Scientific),
Mouse anti-alpha-tubulin (B-5-1-2) (ThermoFisher Scientific),
Goat anti-RNA polymerase II antibody (PLA0292) (Sigma),
Rabbit anti-PCNA antibody (PLA0079 (Sigma),
Mouse anti-BrdU (B44) (1:40) (BD),
Rat anti-BrdU [Bu1/75 (ICR1)] (abcam),
Mouse anti-Mre11 [12D7] ab214 (abcam),
Rabbit anti-biotin (D5A7) (Cell signaling).

Validation

All primary antibodies used in this study have been validated by the manufacturers.

Eukaryotic cell lines

Policy information about cell lines

Cell line source(s)	U2OS and HeLa cells were originally purchased from ATCC. The control and inducible CRISPR/Cas9 RNaseH2A knockout HeLa cell lines were gifts from Dr. Iain Cheeseman whose lab established these cell lines. The TK6 derived lymphoblasts were gifts from Dr. Hiroyuki Sasanuma.
Authentication	HeLa and U2OS cells have the authentication information from ATCC.TK6 derived lymphoblasts were generated by Dr. Sasanuma's lab and identified through genotyping using PCR (ref56). The inducible CRISPR/Cas9 RNaseH2A knockout HeLa cell line was generated by Dr. Cheeseman's lab and we are not aware if authentication was done or not. The gene knockout for Mre11 in TK6 lymphoblasts and RNaseH2A in HeLa cells were validated by western blot in our lab.
Mycoplasma contamination	All cell lines were tested negative for mycoplasma contamination.
Commonly misidentified lines (See ICLAC register)	The study did not involve commonly misidentified lines.

Flow Cytometry

Plots

Confirm that:

☒ The axis labels state the marker and fluorochrome used (e.g. CD4-FITC).

☒ The axis scales are clearly visible. Include numbers along axes only for bottom left plot of group (a 'group' is an analysis of identical markers).

☐ All plots are contour plots with outliers or pseudocolor plots.

☒ A numerical value for number of cells or percentage (with statistics) is provided.

Methodology

Sample preparation	The flow cytometry was performed on TK6 derived lymphocytes indicated above. Cells were harvested, washed with 1XPBS twice, extracted with 25mM HEPES (pH 7.4), 50mM NaCl, 1mM EDTA with protease inhibitor on ice, and fixed with 2% paraformaldehyde in 1x PBS. Cells were then incubated with primary antibody S9.6 (1:200) (millipore) overnight at 4°C and with anti-mouse-IgG-PerCP-Cy5.5 secondary antibody (1:100) (Santa Cruz) for 30 minutes at room temperature. Cells were resuspended in 1XPBS before FACS analysis.
Instrument	FACSCalibur flow cytometer
Software	The data were collected using CellQuestPro software, and analyzed using FlowJo Version 9.3.2.
Cell population abundance	For each replicate, a minimum of 10,000 cells were recorded for every condition.
Gating strategy	Only debris was excluded according to SSC+FSC plot in asynchronized cells.

☐ Tick this box to confirm that a figure exemplifying the gating strategy is provided in the Supplementary Information.
